# The Swedish translation and cross-cultural adaptation of the Functional Assessment of Chronic Illness Therapy – Cervical Dysplasia (FACIT-CD): linguistic validity and reliability of the Swedish version

**DOI:** 10.1186/s12905-017-0381-3

**Published:** 2017-04-04

**Authors:** Marie Rask, Marie Oscarsson, Neil Ludwig, Katarina Swahnberg

**Affiliations:** 1 Department of Health and Caring Sciences, Linnaeus University, SE-391 82 Kalmar, Sweden; 2 FACITtrans, Elmhurst, IL USA

**Keywords:** FACIT-CD, Health-related quality of life (HRQoL), Translation, Cross-cultural adaptation, Linguistic validity, Reliability

## Abstract

**Background:**

Cervical dysplasia is a precancerous condition, which has been shown to create anxiety in women. To be able to investigate these women’s health-related quality of life, a disease-specific instrument is required. There does not seem to be a Swedish version of an instrument to screen for this specific disease. Therefore, this study aims to translate and cross-culturally adapt the Functional Assessment of Chronic Illness Therapy – Cervical Dysplasia (FACIT-CD) into a Swedish context and evaluate its linguistic validity and reliability.

**Methods:**

The Functional Assessment of Chronic Illness Therapy (FACIT) translation methodology was used, which consists of several steps including pilot testing of the FACIT-CD instrument through cognitive debriefing interviews. Ten women diagnosed with cervical dysplasia participated in the cognitive debriefing interviews. The internal consistency reliability of the Swedish FACIT-CD was estimated by Cronbach’s alpha coefficient. Homogeneity of the items was evaluated by corrected item-total correlations. The sample consists of 34 women who were diagnosed with cervical dysplasia.

**Results:**

The translation and cross-cultural adaptation went smoothly without any problems for the majority of the items. The cognitive debriefing interviews indicated that the Swedish FACIT-CD consists of relevant items, is easy to understand and complete, and has unambiguous and comprehensive response categories. The translation and cross-cultural adaptation resulted in a Swedish FACIT-CD, which is conceptually and semantically equivalent to the English version and linguistically valid. The total scale of the Swedish FACIT-CD exhibited good internal consistency reliability with a Cronbach’s alpha coefficient of 0.84, and all of the subscales exhibited acceptable value between 0.71 and 0.81 except the Relationships subscale, which had a value of 0.67. Finally, all but four items exceeded the acceptable level for the corrected item-total correlations of ≥ 0.20.

**Conclusions:**

The Swedish FACIT-CD is conceptually and semantically equivalent to the English version and linguistically valid; further, it exhibits good internal consistency reliability.

## Background

### Cervical dysplasia

Cervical dysplasia is a precancerous condition, and left untreated it can develop into cervical cancer [1]. The major cause of cervical dysplasia is the sexually transmitted infection human papillomavirus (HPV), involved in all of the cervical cancer cases [2]. Cervical dysplasia can be detected through a Papanicolaou (Pap) smear test [3]. In Sweden, annually, approximately 28,000 women receive a Pap smear test result that shows cervical dysplasia, with different degrees of severity [4]. Receiving this result has shown to negatively affect women’s health-related quality of life (HRQoL). The result elicits psychological emotions, such as fear of cancer and worries about fertility [5–11]. In addition, awareness of HPV adds to feelings of stigmatisation [11, 12] as well as blame and anger, and worries about disclosing the test result to others [12]. To be able to investigate these women’s HRQoL in a Swedish context, a valid and reliable disease-specific instrument is required.

In Sweden, previous research assessing the HRQoL associated with cervical dysplasia has used generic instruments such as: State-Trait Anxiety Inventory (STAI), Montgomery-Åsberg Depressive Rating Scale-self rate (MADRS-S) [13, 14] and The Swedish Health Survey Short Form-36 (SF36) [5]. To our knowledge, there is no Swedish disease-specific instrument that assesses the HRQoL associated with cervical dysplasia. However, there is an English disease-specific instrument, the Functional Assessment of Chronic Illness Therapy – Cervical Dysplasia (FACIT-CD) [15].

### Instrument translation and cross-cultural adaptation

Instruments need to be translated and cross-culturally adapted into the language of the research [16]. The process of translation and cross-cultural adaptation of instruments needs to be systematic [17], and a number of different approaches are available for that purpose [16, 18, 19]. The Functional Assessment of Chronic Illness Therapy (FACIT) translation methodology is one approach, with a double-back-translation. The FACIT translation methodology consists of several steps including pilot testing of the instrument with ten patients in the target language. The aim of the FACIT translation methodology is to produce translations of the original source instrument that are equivalent to the source version [20, 21]. The equivalence, in this context, attempts to achieve unbiased equivalent understanding between instruments in different languages, and focuses on conceptual and semantic equivalence [21]. Conceptual equivalence means that the translated items measure the same theoretical constructs as the source, while semantic equivalence refers to the translated items expressing the same meaning as the source [17]. Finally, linguistic validation is the entire process for the translation and cross-cultural adaptation of the instrument into the target language, in such a way that the instrument remains conceptual and semantic equivalence with the source version [19].

### Functional Assessment of Chronic Illness Therapy – Cervical Dysplasia (FACIT-CD)

The FACIT-CD was developed in 2010 [15] according to the FACIT measurement system [22]. FACIT-CD is a self-administered instrument that takes 10–15 min to complete, and it is designed to assess the HRQoL (based on the past 7 days) associated with cervical dysplasia. The instrument consists of 36 items divided into five subscales: Physical well-being (8 items), Treatment satisfaction (4 items), General perceptions (7 items), Emotional well-being (11 items) and Relationships (6 items). All items use Likert-type response categories, ranging from ‘0’ = ‘not at all’ to ‘4’ = ‘very much’, with the exception of two items that have response categories Yes/No. Negatively worded items are reverse-scored, and all items are summed to create a single score, between a range from 0 to 136, with high scores indicating better HRQoL [15]. The FACIT-CD is available at www.facit.org/

This study was undertaken because of an upcoming interventions study, which advantageously uses a disease-specific instrument that assesses HRQoL associated with cervical dysplasia. We considered the FACIT-CD as an appropriate instrument because: (1) the instrument is disease-specific for cervical dysplasia; (2) the instrument contains a broad set of domains of HRQoL and (3) the instrument was developed using a multi-step rigorous process [15]. The aim of this study was to translate and cross-culturally adapt the Functional Assessment of Chronic Illness Therapy – Cervical Dysplasia (FACIT-CD) into a Swedish context and evaluate its linguistic validity and reliability.

## Methods

The method section consists of translation and cross-cultural adaptation, pilot test (which is one of the steps in the translation and cross-cultural adaptation) and evaluation of the reliability.

### Translation and cross-cultural adaptation

The translation and cross-cultural adaptation of the English FACIT-CD into Swedish was conducted according to the FACIT translation methodology [20, 21]. A document called the Swedish Item History (SWE IH) was used to document the steps of the translation and cross-cultural adaptation process as well as the results of every step. Seven persons were involved; three forward translators, one reconciler, one back translator, one reviewer—also called the language coordinator (the first author)—and the FACIT project manager (the third author). Two of the forward translators, the reconciler and the reviewer/language coordinator were native Swedish speakers and healthcare professionals. The third forward translator was a native English speaker who works as a professional translator. The back translator and the FACIT project manager were both native English speakers.

**Fig. 1:**
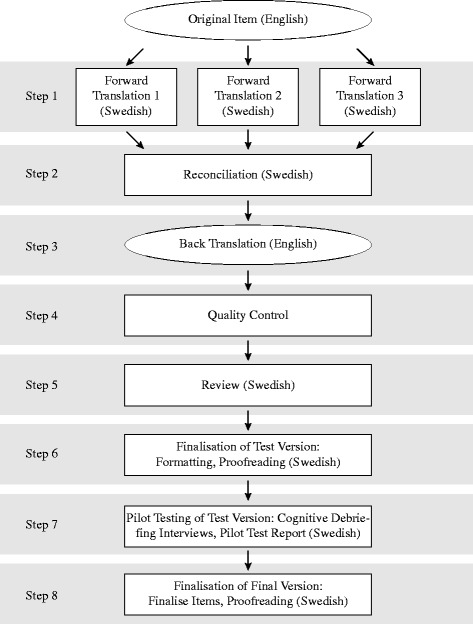
A flowchart of the Functional Assessment of Chronic Illness Therapy (FACIT) translation methodology by Eremenco et al. [21]. Modified by the first author (MR)

In accordance with the FACIT translation methodology, the steps described below were followed. (A flowchart of the FACIT translation methodology is outlined in Fig. 1). Beginning with forward translations, the three translators independently presented translations of the English version into Swedish, with a focus on capturing the meaning of the items. The second step was the reconciliation, by the reconciler to resolve discrepancies between the forward translations, using the English version as reference. This was done by combining them, or providing an alternative translation, with the aim to capture the meaning of the source while not straying too far from the English words or structure. In the third step, back translation, the back translator, who had no access to the forward translations or the original English version, translated the reconciled translations to English, capturing the literal meaning of the translation. In the fourth step, quality control, the SWE IH document, consisting of the forward translations, the reconciled and the back translations, was sent to the FACIT project manager. The items’ back translations were compared with the original English source, and any thoughts and/or concerns were expressed. The fifth step was the review process, where the reviewer/language coordinator analysed the forward translations, reconciliation and back translations as well as the comments expressed by the FACIT project manager. Response commentary was provided, and the reconciled translations were either modified or used completely to form a test version of the translation. In step six, finalisation of the test version, the FACIT project manager evaluated the completed reviewer assessments and final translations, and then communicated any remaining or new concerns to the reviewer/language coordinator. The resolution resulted in a test version that was formatted into the instrument and then proofread for final grammatical, spelling and formatting errors by the reviewer/language coordinator and one of the forward translators.

The seventh step consisted of pilot testing the test version of the instrument in the target language through ten cognitive debriefing interviews (further described later in the method section). These interviews allowed the FACIT project manager and the reviewer/language coordinator to assess specific characteristics of the items’ translation, as well as themes exhibited in the comments. This provided evidence to confirm that translations had been accurately understood in relation to the intended meaning as defined by the item’s definition. By asking about the instrument’s phrasing, ease of comprehensibility and general relevance, the FACIT project manager was able to empirically analyse data pertaining to the linguistic validity and acceptability of the instrument. The analyses of the data from the cognitive debriefing interviews resulted in a pilot test report. In the eighth step, the finalisation of the final version, items were finalised as is or changed based on the pilot test report results, and a final validated version of the translated instrument was created. This final version was proofread for grammatical, spelling and formatting errors by the reviewer/language coordinator and one of the forward translators. The translation and cross-cultural adaptation of the FACIT-CD occurred between January and October 2015.

### Pilot test and evaluation of the reliability

#### Participants and recruitment for the pilot test and evaluation of the reliability

Two different samples were used; one for the pilot test (*n* = 10), and one for evaluation of the reliability (*n* = 35), although one woman was excluded since the entire FACIT-CD was not completed (*n* = 34). A sample of 15–30 women was considered to be sufficient for evaluation of the reliability [21]. The inclusion criteria were: aged ≥ 18 years, diagnosed with cervical dysplasia and having Swedish as native language (the latter only for the pilot test). Women were recruited at a women’s health clinic in a rural district of south-eastern Sweden, while attending a follow-up of their cervical dysplasia. The manager at the clinic was contacted by the first author and informed about the study. Midwives responsible for the women attending the follow-up were designated to give the women oral and written information about the study and invite them to participate, and this applied to both samples. First, one sample of women were invited to participate in the pilot test. Those who accepted provided their names and phone numbers to the midwife who forwarded the information to the first author. The first author booked the time and place for the pilot test in consultation with the women. Secondly, women were recruited for evaluation of the reliability. Those who accepted to participate were referred to the nurse at the clinic who was responsible for the data collection for this part of the study.

#### Data collection for the pilot test

Data were collected through cognitive debriefing interviews, which were performed using a test packet, one for each woman. The test packet included the Swedish test version of the FACIT-CD and the semi-structured patient interview form (PIF). These two documents were paired using a code. The women received the Swedish FACIT-CD and were asked to complete it. After completion, the individual face-to-face semi-structured cognitive debriefing interviews were performed using the PIF. The interview form consisted of socio-demographic questions (*n* = 3) and general questions about the Swedish FACIT-CD, as well as questions on each item (*n* = 84). Example of a general question was: ‘Were there any items that were difficult to understand? If so, would you please tell me which items and why they were difficult?’ Examples of questions on specific items were: I will now ask you some questions about item CD5: I worry about spreading the infection. ‘Which response did you choose and why?’ and ‘What does the word “worry” mean to you in this item?’ The interview technique used was both think-aloud and verbal probing according to Willis [23].

The interviews lasted between 43 and 90 min, and nine of them took place in the women’s home while one was at a university. All interviews were recorded and typed into the PIF in Swedish, but also in English as translated by the first author. The PIFs were sent in a secure file to the FACIT project manager for further analyses. Data were collected in June 2015.

#### Data analysis for the pilot test

The cognitive debriefing interviews were analysed by the FACIT project manager, who determined if the comments were of a conceptual, semantic or stylistic nature. The FACIT project manager’s concerns regarding the women’s comments were presented to the reviewer/language coordinator (who was the interviewer) to provide further input and resolve queries regarding why women may have responded in a certain way. The reviewer/language coordinator’s input was further reviewed, and in some cases investigated further, until consensus was reached for each item.

The socio-demographic characteristics of the women in the sample were summarised by descriptive statistics (absolute frequency, mean and range).

#### Data collection for evaluation of the reliability

Data were collected using a questionnaire, which consisted of six socio-demographic questions and the Swedish FACIT-CD. The nurse at the clinic responsible for the data collection asked the women to complete the questionnaire. All of the women completed the questionnaire anonymously, in a secluded place at the women’s health clinic, when they were attending a follow-up appointment for their cervical dysplasia. The completed questionnaires were then retrieved by the first author. Data were collected between October 2015 and January 2016.

#### Data analysis for evaluation of the reliability

The internal consistency reliability of the total scale as well as of each subscale was estimated by Cronbach’s alpha coefficient. Values ≥ 0.70 were considered acceptable, ≥ 0.80 was considered good and ≥ 0.90 was excellent [24]. Homogeneity of the items was evaluated by corrected item-total correlations, to identify items with poor correlations with the respective subscale. An acceptable level of the corrected item-total correlations was set to ≥ 0.20 [21]. All statistical analyses were conducted using SPSS for Windows statistical software version 21 (SPSS, Inc., Chicago, IL, USA).

The socio-demographic characteristics of the women in the sample were summarised by descriptive statistics (absolute frequency, mean and range).

## Results

The result of the translation and cross-cultural adaptation was a Swedish FACIT-CD that is conceptually and semantically equivalent to the English version and is linguistically valid. See Tables 1, 2, 3, 4 and 5 for the results of the translation and cross-cultural adaptation of each item. The translation and cross-cultural adaptation and the pilot test are presented separately.

**Table 1:** Results of translation and cross-cultural adaptation, and Cronbach’s alpha coefficient, and corrected item-total correlation for the Swedish FACIT-CD subscale Physical well-being (*n* = 34)

Item code	The original source item and the translated item	α	Corrected item-total correlation	α if item deleted
	Physical well-being/Fysiskt välbefinnande	0.71		
CD1	I have discomfort in my pelvic area			
	Jag känner obehag i mitt underliv		0.78	0.59
CD2	I have pain in my pelvic area			
	Jag känner smärta i mitt underliv		0.55	0.65
CD3	I have cramping in my pelvic area			
	Jag har kramper i mitt underliv		0.46	0.68
Cx1	I am bothered by discharge or bleeding from my vagina			
	Jag besväras av flytningar eller blödningar från slidan		0.34	0.71
GP5	I am bothered by side effects of treatment			
	Jag besväras av biverkningar av behandlingen		0.11^a^	0.73
ES8	I have pain or discomfort with intercourse			
	Jag känner smärta eller obehag vid samlag		0.55	0.65
CD4	I have to limit my sexual activity because of the infection			
	Jag måste begränsa min sexuella aktivitet på grund av infektionen		0.42	0.68
CD5	I worry about spreading the infection			
	Jag oroar mig för att sprida infektionen		0.08^a^	0.75

^a^Corrected item-total correlation below the acceptable level of ≥ 0.20

**Table 2:** Results of translation and cross-cultural adaptation, and Cronbach’s alpha coefficient, and corrected item-total correlation for the Swedish FACIT-CD subscale Treatment satisfaction (*n* = 34)

Item code	The original source item and the translated item	α	Corrected item-total correlation	α if item deleted
	Treatment satisfaction/Tillfredställelse med behandling	0.81		
GR1	I have confidence in my doctor(s)			
	Jag har förtroende för min(a) läkare		0.55	0.81
CD6	I feel that I received the treatment that was right for me			
	Jag känner att jag har fått den behandling som var rätt för mig		0.69	0.74
CD7	My doctor gave me explanations that I could understand			
	Min läkare gav mig förklaringar (information) som jag kunde förstå		0.79	0.68
CD8	My doctor explained the possible benefits of my treatment			
	Min läkare förklarade de eventuella fördelarna med min behandling		0.62	0.81

**Table 3:** Results of translation and cross-cultural adaptation, and Cronbach’s alpha coefficient, and corrected item-total correlation for the Swedish FACIT-CD subscale General perceptions (*n* = 34)

Item code	The original source item and the translated item	α	Corrected item-total correlation	α if item deleted
	General perceptions/Allmänna uppfattningar	0.74		
GF1	I am able to work (include work at home)			
	Jag kan arbeta (innefattar även arbete i hemmet)		0.13^a^	0.76
GF3	I am able to enjoy life			
	Jag kan njuta av livet		0.47	0.72
HI11	I am hopeful about the future			
	Jag är hoppfull inför framtiden		0.62	0.70
Sp9	I find comfort in my faith or spiritual beliefs			
	Jag finner tröst i min tro eller inre övertygelse		0.41	0.75
GF7	I am content with the quality of my life right now			
	Jag är nöjd med min livskvalitet just nu		0.57	0.69
CD9	I feel that I can manage things that come up around this infection			
	Jag känner att jag kan hantera saker som dyker upp runt den här infektionen		0.67	0.65
CD10	I have accepted that I have this infection			
	Jag har accepterat att jag har den här infektionen		0.57	0.68

^a^Corrected item-total correlation below the acceptable level of ≥ 0.20

**Table 4:** Results of translation and cross-cultural adaptation, and Cronbach’s alpha coefficient, and corrected item-total correlation for the Swedish FACIT-CD subscale Emotional well-being (*n* = 34)

Item code	The original source item and the translated item	α	Corrected item-total correlation	α if item deleted
	Emotional well-being/Känslomässigt välbefinnande	0.79		
CD11	I worry that the infection will get worse			
	Jag oroar mig för att infektionen kommer bli värre		0.39	0.78
CD12	I have hidden this problem so others will not notice			
	Jag har dolt detta problem så att andra inte kommer att märka det		0.48	0.77
CD13	I have concerns about my ability to become pregnant			
	Jag är bekymrad över min förmåga att bli gravid		0.40	0.78
BMT18	The cost of my treatment is a burden on me or my family			
	Kostnaden för min behandling är en börda för mig eller min familj		0.42	0.77
CD14	I worry about other people’s attitudes towards me			
	Jag oroar mig för andra människors attityder till mig		0.42	0.77
CD15	I feel embarrassed about the infection			
	Jag känner mig generad över infektionen		0.65	0.75
CD16	I tend to blame myself for the infection			
	Jag har en tendens att klandra mig själv för infektionen		0.68	0.74
CD17	I was careful who I told about the infection			
	Jag var försiktig med för vem jag berättade om infektionen		0.34	0.78
CD18	I have had difficulty telling my partner/spouse about the infection			
	Jag har haft svårt att berätta för min partner/make om infektionen		0.36	0.78
CD19	I am frustrated by the infection			
	Jag är frustrerad över infektionen		0.49	0.77
CD20	I am depressed about the infection			
	Jag är nedstämd av infektionen		0.39	0.78

**Table 5:** Results of translation and cross-cultural adaptation, and Cronbach’s alpha coefficient, and corrected item-total correlation for the Swedish FACIT-CD subscale Relationships (*n* = 34)

Item code	The original source item and the translated item	α	Corrected item-total correlation	α if item deleted
	Relationships/Relationer	0.67^a^		
Q9	I have told my partner/spouse about my infection		^b^	^b^
	Jag har berättat för min partner/make om min infektion		^b^	^b^
CD21	I get emotional support from my partner/spouse			
	Om ja: Jag får känslomässigt stöd från min partner/make		0.66	0.43
Q10	I have told family members about my infection		^b^	^b^
	Jag har berättat för familjemedlemmar om min infektion		^b^	^b^
CD22	I get emotional support from family members			
	Om ja: Jag får känslomässigt stöd från familjemedlemmar		0.75	0.35
GS1	I feel close to my friends			
	Jag känner närhet till mina vänner		0.01^c^	0.82
HI3	I have people to help me if I need it			
	Det finns personer som hjälper mig om jag behöver det		0.67	0.58

^a^Cronbach’s alpha coefficient below the acceptable level of ≥ 0.70; ^b^Have the response options Yes/No, Ja/Nej; ^c^Corrected item-total correlation below the acceptable level of ≥ 0.20

The result section ends with evaluation of the reliability, which indicated that the Swedish FACIT-CD exhibited good internal consistency reliability of the total scale, and that the majority of the items in the respective subscales exhibited acceptable corrected item-total correlations.

### Translation and cross-cultural adaptation

The translation and cross-cultural adaptation went smoothly without any problems, except for the three items that consisted of the term “pelvic area” (CD1, CD2 and CD3). Initially, this term was translated by two of the forward translators and the reconciler into Swedish as “bäckenområde”. However, these three individuals had no previous professional contact with women who had been diagnosed with cervical dysplasia. The remaining forward translator was a midwife and familiar with this type of diagnoses, and she translated the term “pelvic area” into Swedish as “underliv”.

During the translation and cross-cultural adaptation process, the Swedish terms “bäckenområde” and “underliv” were discussed extensively between the FACIT project manager and the reviewer/language coordinator. The latter strongly felt that the term “underliv” was the preferable term in the context of cervical dysplasia. The FACIT project manager preferred the Swedish term “bäckenområde”, since it was a more literal translation of the “pelvic area”, closer to the source. As a result, the test version was finalised with the term “bäckenområde”, but the term “underliv” was used as an alternative translation, discussed in the cognitive debriefing interviews. The interviews determined which Swedish term should be used.

Additionally, one item (Sp9) changed the source word “spiritual beliefs” in Swedish to use “inre övertygelse”, which could be back translated into English as “inner beliefs”. This change was based on additional review in a separate project from this study, and during the same time period.

Finally, the result of the translation and cross-cultural adaptation process suggests that the Swedish FACIT-CD is conceptually and semantically equivalent to the English version and linguistically valid.

### Pilot test

#### Women’s socio-demographic characteristics

In total, ten women were interviewed. The mean age was 33 years (range 25–46 years). All of the women were native Swedish speakers and none were receiving any treatment at the time of the interviews. The average time between when the women were diagnosed and when the interviews took place was 8 months (range 1–18 months).

#### Cognitive debriefing interviews

The women displayed good understanding of the items, and the responses they selected corresponded with the reasons they provided for choosing those answers. According to the women, all of the items were relevant to their diagnosis, the instructions were easy to understand and the response categories were unambiguous and comprehensive. The women also reported that the Swedish FACIT-CD was easy to complete in general.

Results from the cognitive debriefing interviews led to some changes in eight of the items in the Swedish FACIT-CD. The items CD1, CD2 and CD3 were changed to use the term “underliv”. One of the items had the Swedish word “information” added in parentheses (CD7). Four of the items had structural change, where the phrase “if yes” was moved from the question introduction (Q9 and Q10) and placed instead before the respective follow-up questions (CD21 and CD22).

### Evaluation of the reliability

#### Women’s socio-demographic characteristics

In total, 34 women completed the Swedish FACIT-CD without skipping data. The mean age was 36 years (range 23–64 years). Of these women, 14 had completed senior high school and 16 college or higher education. Twenty-four women had a partner, and 20 had one or more children. One of the women was pregnant at the time that she completed the Swedish FACIT-CD. Three of the women were born abroad, either in Kosovo, Spain or Thailand, and all of the other women were Swedish born. All the women completed the questionnaire within a year after the diagnosis.

#### Internal consistency reliability and homogeneity

The total scale of the Swedish FACIT-CD exhibited good internal consistency reliability with a Cronbach’s alpha coefficient of 0.84. Cronbach’s alpha coefficient was acceptable on four out of five of the subscales: Physical well-being α = 0.71, Treatment satisfaction α = 0.81, General perceptions α = 0.74 and Emotional well-being α = 0.79. Nevertheless, the subscale Relationships had a Cronbach’s alpha coefficient of 0.67, which is below the acceptable value of ≥ 0.70. The corrected item-total correlations for each subscale are shown in Tables 1, 2, 3, 4 and 5. The majority of the items exhibited acceptable corrected item-total correlations, with the following exceptions in the respective subscales: Physical well-being subscale 0.11 (if item deleted α = 0.73) and 0.08 (if item deleted α = 0.75), Relationships subscale 0.01 (if item deleted α = 0.82) and General perceptions subscale 0.13 (if item deleted α = 0.76).

## Discussion

The purpose of this study was to translate and cross-culturally adapt the Functional Assessment of Chronic Illness Therapy – Cervical Dysplasia (FACIT-CD) into a Swedish context, and evaluate the linguistic validity and reliability. To the best of our knowledge, the FACIT-CD is the first Swedish disease-specific instrument that assesses HRQoL associated with cervical dysplasia. The results indicated that the Swedish FACIT-CD is conceptually and semantically equivalent to the English version and linguistically valid; further, it exhibits good internal consistency reliability.

In this study, the translation and cross-cultural adaptation went smoothly without any problems for the majority of the items. One reason may be because the FACIT-CD is developed in the US [15], and American and Swedish cultures seem to be relatively similar; additionally, they are both Germanic languages. This facilitates the possibility to reach semantic and conceptual equivalence between the original and the target version of the instrument. It is essential to reach semantic and conceptual equivalence, as it is the basis for determining whether an instrument can be adapted to the target culture [25]. Although it is impossible to attain 100% equivalence, it is essential to strive to minimise bias and attain as strong equivalence as possible [21]. In order to increase the possibility of attaining strong equivalence, it is essential to use a rigorous translation and cross-cultural adaptation method [16]. In this study, the FACIT translation methodology was used, which seems to be a more rigorous version of the double-back-translation method [26]. The FACIT translation methodology is considered more rigorous because it has a multi-step approach, including cognitive debriefing interviews in the target language [21]. Furthermore, the methodology provided opportunities for dialogue between the reviewer/language coordinator and the FACIT project manager, where the items were discussed and further probed. This allowed us to ensure appropriate decision-making regarding the translation and cross-cultural adaptation of each item. This study encountered specific issues during the translation and cross-cultural adaptation process. There were some discussions between the FACIT project manager and the reviewer/language coordinator regarding which Swedish term should be used for the term pelvic area in three of the items. These items were debriefed in the cognitive interviews, which provided information to clarify which Swedish term should finally be used in the FACIT-CD. The cognitive debriefing interviews were a very important step in finalising items in the translation and cross-cultural adaptation process.

After an instrument has been translated and cross-culturally adapted, it is highly recommended to statistically evaluate that version produced [16]. Accordingly, statistical analyses were performed on the Swedish FACIT-CD. With regard to the internal consistency reliability of the FACIT-CD subscales, the Cronbach’s alpha assessment demonstrated acceptable (α coefficients ≥ 0.70) to good (α coefficients ≥ 0.80) internal consistency reliability, except for the Relationships subscale, which had a somewhat low Cronbach’s alpha coefficient (0.67) [24]. This low value for the Relationships subscale could be explained by the fact that the item *I feel close to my friends* (GS1) exhibited significantly low corrected item-total correlation of 0.01. Our findings suggest that the Relationships subscale could be improved by deleting the item GS1 (deleted α = 0.82), as it did not seem to be measuring the same construct as the other items. Furthermore, each of the Physical well-being and General perceptions subscales had at least one item, respectively, which demonstrated significantly low corrected item-total correlation: specifically, 0.11 on item *I am bothered by side effects of treatment* (GP5) and 0.13 on item *I am able to work (include work at home)* (GF1). Nevertheless, even with the items deleted, none of them dramatically increased the respective subscales’ value of Cronbach’s alpha. The lack of studies evaluating the psychometric properties of the FACIT-CD made it impossible to perform some comparisons. However, the items mentioned above have previously been psychometrically evaluated in a study [27] with samples from five Spanish-speaking countries using the Functional Assessment of Cancer Therapy – Gastric Cancer (FACT-Ga). In all of the Spanish countries, these items demonstrated corrected item-total correlations over the acceptable value of ≥ 0.20 [21]. However, one of the Spanish countries demonstrated low corrected item-total correlation on the item *I feel close to my friends* (GS1), and two countries on the item *I am able to work (include work at home)* (GF1) [27], which could correspond to our results. Attention must be paid to the fact that the FACIT-CD and FACT-Ga subscales differ, which affects the corrected item-total correlation in the respective subscales. Furthermore, the Physical well-being subscale also demonstrated a corrected item-total correlation of 0.08 for the item *I worry about spreading the infection* (CD5), but if the item was deleted, the Cronbach’s alpha coefficient increased to 0.75. In order to improve the physical well-being subscale, our findings suggest that this item might be deleted unless there are theoretical reasons for not doing so [17]. However, this item relates to a physical situation, about spreading the disease, which does not necessarily reflect on the emotional element about how you feel about yourself or your situation.

There were some limitations to this study. One possible limitation is related to the small sample size (*n* = 34) regarding the evaluation of the internal consistency reliability. However, according to Eremenco [21], a sample size of 15–30 is sufficient to provide preliminary evidence for internal consistency reliability using Cronbach’s alpha. Another limitation might be that the cut-off at ≥ 0.20 is used for an acceptable corrected item-total correlation, while the most common cut-off is ≥ 0.30 [24]. However, we followed the FACIT translation methodology recommendation of the cut-off at ≥ 0.20, according to Eremenco [21]. Furthermore, the stability of the Swedish FACIT-CD was not evaluated, as no data were collected a second time.

Further studies are recommended to conclude that the Swedish FACIT-CD is valid and reliable with a larger sample, and this is warranted before any decision on whether or not to delete the items discussed above. To confirm the original factor structure of the Swedish FACIT-CD, an exploratory factor analyses can be used. Finally, we plan to use the Swedish FACIT-CD in an intervention study with a larger sample.

## Conclusions

The results from this study indicate that the Swedish Functional Assessment of Chronic Illness Therapy – Cervical Dysplasia (FACIT-CD) is conceptually and semantically equivalent to the English version and linguistically valid; moreover, it has good internal consistency reliability. Furthermore, the Swedish FACIT-CD consists of relevant items, is easy to understand and complete, and has unambiguous and comprehensive response categories. However, evaluations of the Swedish FACIT-CD’s psychometric proprieties are recommended.

## Abbreviations

FACIT: Functional assessment of chronic illness therapy
FACIT-CD: Functional assessment of chronic illness therapy – cervical dysplasia
HRQoL: Health-related quality of life
PIF: Patient interview form
SWE IH: Swedish item history
